# The Bioprotective Effects of Marigold Tea Polyphenols on Obesity and Oxidative Stress Biomarkers in High-Fat-Sugar Diet-Fed Rats

**DOI:** 10.1155/2024/3833521

**Published:** 2024-10-04

**Authors:** Bader Alsuwayt, Neelam Iftikhar, Abdullah Ijaz Hussain, Ashfaq Ahmad, Irsa Zafar, Arifa Khanam, Wen-Nee Tan, Lutfun Nahar, Afaf F. Almuqati, Esraa Mohammad Haji, Ali F. Almutairy, Satyajit D. Sarker

**Affiliations:** ^1^Department of Pharmacy Practice, College of Pharmacy, University of Hafr Al Batin, Hafr Al Batin 39524, Saudi Arabia; ^2^Department of Chemistry, Government College University Faisalabad, Faisalabad 38000, Pakistan; ^3^Chemistry Section, School of Distance Education, Universiti Sains Malaysia, Penang 11800, Malaysia; ^4^Laboratory of Growth Regulators, Institute of Experimental Botany ASCR & Palacký University, Šlechtitelů 27 78371, Olomouc, Czech Republic; ^5^Department of Pharmaceutical Chemistry, College of Pharmacy, University of Hafr Al Batin, Hafr Al Batin 39524, Saudi Arabia; ^6^Department of Pharmacology and Toxicology, College of Pharmacy, Qassim University, Buraydah 51452, Saudi Arabia; ^7^Centre for Natural Products Discovery, School of Pharmacy and Biomolecular Sciences, Liverpool John Moores University, James Parsons Building, Byrom Street, Liverpool L3 3AF, UK

**Keywords:** lipid profile, marigold petal tea, obesity, oxidative stress, rutin

## Abstract

**Background:** The research is aimed at exploring the potential of marigold petal tea (MPT), rich in polyphenol contents, against oxidative stress and obesity in a rat model following a high-fat-sugar diet (HFSD).

**Methods:** The MPT was prepared through the customary method of decoction and was subjected to analysis for its polyphenol composition using reversed-phase high-performance liquid chromatography (RP-HPLC). Two specific doses of MPT, namely, 250 and 500 mg/kg body weight (BW), were chosen for the study—referred to as MPT-250 and MPT-500, respectively.

**Result:** The main phenolic acids and flavonoids identified in MPT, with concentrations exceeding 10 mg/100 mL of tea, included catechin, rutin, salicylic acid, gallic acid, sinapic acid, chlorogenic acid, cinnamic acid, and ellagic acid. The total phenolic (TP) and total flavonoid (TF) contents in MPT were measured to be 5.53 and 7.73 mg/g, respectively. Additionally, MPT demonstrated a 57.2% scavenging capacity with 2,2-diphenyl-1-picrylhydrazyl radical. Notably, the administration of a higher dose (MPT-500) showed a significant reduction in body mass index (BMI) and a 51.24% reduction in the rate of increase in BW compared to the HFSD group. The findings indicated that all the treatment groups, that is, orlistat treatment (OT), MPT-250, and MPT-500 groups, experienced reduced levels of serum total cholesterol (TC), triglyceride (TG), and markers of lipoproteins in contrast to the HFSD group. Moreover, MPT helped restore the levels of malondialdehyde (MDA), superoxide dismutase (SOD), and reduced glutathione (GSH), thereby demonstrating its potential in combating oxidative stress. The MPT-500 group also displayed decreased liver and kidney weights and an improved atherogenic index when compared to the HFSD group.

**Conclusion:** The results clearly indicate that a high dosage of MPT showed antiobesity activity which was comparable to the same effects produced by the conventional drug orlistat.

## 1. Introduction

Obesity and excessive weight gain have become significant public health concerns in the 21st century, impacting people of all age groups. These issues are associated with a wide range of complications, including various cancers, chronic heart diseases, Type 2 diabetes, metabolic syndromes, joint problems, and psychological and social effects [1–4]. In adipocytes, the excess storage of triglycerides (TGs) can cause obesity and increased TG levels in the liver, blood, and muscle tissues of overweight individuals which cause pathological disorders [5, 6]. Body mass index (BMI) is a universal representation of obesity and overweight. For instance, a BMI of 25 is regarded as overweight, while a BMI of 30 is considered obesity. When BMI is 40 or more, morbid obesity is referred to as Type III obesity and causes more complications [7]. Obesity can be considered among the Top 5 risk factors that can cause death that reaches approximately 2.8 million yearly [8, 9].

Obesity is correlated with augmented oxidative stress which later increases the production of free radicals and reactive oxygen species (ROS) [10]. These ROS may come from the mitochondrial respiratory chain and the nicotinamide adenine dinucleotide phosphate (NADPH) oxidase, but obesity can induce oxidative stress on its own [10]. The prevalence of obesity is influenced by a combination of genetic, lifestyle, and dietary factors [11, 12]. Other reasons for this increase in weight gain tendency are steroid hormones and psychoactive drugs [2].

Orlistat is a commonly prescribed medication for addressing persistent obesity and has received approval from the European Medicines Agency (EMA). Acting as an inhibitor of pancreatic and gastric lipases, it can effectively hinder the absorption of approximately 25%–30% of the calories derived from ingested fat [13]. Nevertheless, its use has been linked to certain adverse effects, and its pharmacological impact is contingent upon patients adhering to specific dietary constraints, thus limiting its overall effectiveness [13, 14].

Recently, plant-based natural products are gaining the attention of health practitioners as protective and therapeutic strategies in many ailments including obesity [15]. Polyphenols are employed against oxidative stress and associated diseases [15, 16]. Therefore, polyphenols are used as antiobesity due to their safety profile, affordability, and significant efficacy [15]. Herbal local teas which include herbal infusions like Gurhal, Ginger, Podina, Eliechi, and Genda teas are the most consumed beverages [16–18]. They are consumed either as refreshment drinks or to reduce body weight (BW). This is mainly due to fewer side effects as compared to pharmacological drugs, thus gaining much recognition [19, 20].

In the present work, marigold petal tea (MPT) was selected to investigate its antioxidant and antiobesity potentials. The identification and quantification of polyphenols were carried out by reversed-phase high-performance liquid chromatography (RP-HPLC). The antioxidant potential of MPT was estimated by various antioxidant assays. Furthermore, the antiobesity potential of MPT with a high-fat-sugar diet (HFSD)-induced obesity model was determined.

## 2. Materials and Methods

### 2.1. Plant Materials, Chemicals, and Reagents

The petals of marigold (*Calendula officinalis* L.) were collected from the botanical garden and identified by the local taxonomist of Government College University Faisalabad (GCUF), Pakistan. All the standards employed in the chromatographic analysis were of high-performance liquid chromatography (HPLC) grade and procured from Sigma Chemical Co. (St. Louis, MO, United States).

### 2.2. Preparation of MPT

The marigold petals were washed with deionized water, dried in a dehydrator, and grinded (80 meshes). The preparation of MPT involved the use of a temperature-controlled orbital shaker (Gallenkamp, UK). Twenty grams of powdered sample was subjected to shaking in a volume of 200 mL of distilled water at 50°C for 24 h. The solid residues were filtered, while the resulting solution was dried utilizing a rotary evaporator as reported in the previous study [21]. The percentage yield was computed using the following formula.

 $$\begin{array}{c} \text{Yield}\left(\frac{\text{g}}{100\text{g}}\right)=\frac{\text{Weight of dry extract}}{\text{Weight of dry plant material}}\times 100 \end{array}$$

### 2.3. Chromatographic Analysis of MPT for Phenolic Acids and Flavonoids

The HPLC was utilized for quantitative assessment of phenolic acids and flavonoids from MPT as previously documented [22]. For this purpose, a Flexar HPLC system equipped with a gradient model Flexar pump, LC-Shelton CT 06484, and a UV/visible detector were selected (Perkin Elmer, United States). To achieve chromatographic separation, a hypersil GOLD C_18_ column measuring 250 × 4.6 mm with a particle size of 5 *μ*m (Thermo Fisher Scientific Inc.) was used. The quantification of phenolic acids and flavonoids was executed using a standard addition method.

### 2.4. In Vitro Antioxidant Activity of MPT

The total phenolic content (TPC) of the MPT was measured by using the Folin–Ciocalteu reagent method, as detailed in a previous study [21]. A standard curve ranging from 10 to 80 ppm of gallic acid was established to compute the TPC of the sample, which was measured as equivalence (*y* = 0.026*x* + 0.000, *R*_2_ = 0.997) to gallic acid. Additionally, the total flavonoid contents (TFCs) of the MPT were assessed using a spectrophotometric method outlined [21]. To determine the TFC of the sample in terms of milligrams of flavonoids per gram of plant material, a standard curve was prepared using concentrations ranging from 10 to 160 ppm of catechin, measured as catechin equivalent (*y* = 0.006*x* + 0.015, *R*2 = 0.999). Furthermore, 2,2-diphenyl-1-picrylhydrazyl (DPPH) free radical scavenging activity of the MPT was carried out following the methodology described [23]. A solution of tea (10 *μ*g/mL) and standard butylated hydroxytoluene (BHT) were mixed with 2 mL of 90 *μ*M DPPH solutions in methanol, and the absorbance was taken at 517 nm after 30 min.

### 2.5. In Vivo Antiobesity Activity

#### 2.5.1. Animal Recruited for Study

Thirty young male Wistar Kyoto (WKY) rats having 140–155 g weight were brought to the animal house and kept at the standard facility of the animal house in GCUF, Pakistan. Six rats were picked randomly and distributed in each labeled cage (16 × 13.5 × 6.25 in.) for each group, and we provided them with standard food. Approval of the study (study no. 19680/IRB no. 680) for experiments on animals was performed according to the guidelines provided by the Institutional Review Board for Animal Studies, GCUF, Faisalabad, Pakistan.

#### 2.5.2. Composition and Content of HFSD

For the in vivo experiment, two types of diets were formulated: a standard diet consisting of rat chow and a HFSD. The standard diet comprised cellulose (5%), corn starch (50%), corn oil (5%), casein (26%), sucrose (9%), mineral mixture (4%), and vitamin mixture (1%). The high HFSD consisted of cellulose (5%), starch from corn (15%), casein content (26%), beef tallow content (40%), sucrose (9%), mixtures of minerals (4%), and vitamins (1%). Additionally, the animals were supplemented with a carbonated soft drink solution in a 1:1 ratio with water. Notably, the high-fat diet exhibited a higher lipid content, resulting in an expected energy discrepancy of 4.37 KJ/g. The diets were securely stored in airtight bags at 24°C in a dark environment to prevent the oxidation of fat.

#### 2.5.3. Experimental Design

After a 7-day acclimatization period, the 30 healthy rats were selected and randomly divided into the following five groups: normal control (NC), HFSD, orlistat-treated (OT), marigold petal tea-250 (MPT-250), and marigold petal tea-500 (MPT-500) groups. The NC group provided a normal diet (daily intake of 20 g/rat) and water ad libitum, and the HDSD group provided HFSD (approximately 20 g/rat/day) and carbonated drink ad libitum. Treatment groups OT, MPT-250, and MPT-500 were supplied with orlistat 250 mg/kg BW, MPT 250 mg/kg BW, and MPT 500 mg/kg BW for 28 days along with HFSD (approximately 20 g/rat/day). The orlistat or MPT doses were administered to treatment groups by mouth via oral gavage.

#### 2.5.4. Measurement of BW and BMI

The weight of each individual rat was measured both at the beginning and end of the study, and the change in BW for each group was determined using the BMI calculation. The BMI was calculated by dividing the weight (gram) by the length (square centimeter) [22] taken as an obesity indicator [24].

#### 2.5.5. Collection of Blood Samples and Body Organs

At the end of the experiment, rats were subjected to an overnight fast but were provided free access to water. Blood samples were obtained by following the procedure reported previously [25]. Briefly, 4 mL of blood was taken from the right carotid artery, under chloroform anesthesia, into a tube and centrifuged for 15 min at 3000 rpm. The clear layer of serum was transferred into microcentrifuge tubes, labeled, and stored at −70°C until biochemical investigation. Upon completion of blood collection, the animals were euthanized by exsanguinations under chloroform anesthesia. The kidneys and liver were quickly extracted, cleaned, rinsed with normal saline, and weighed. These organs were then preserved in 10% formalin for subsequent histological examination. To assess organ health and size, the kidney index and liver index were calculated as reported [26].

### 2.6. Biochemical Investigations

#### 2.6.1. Estimation of Lipid Profile

Total cholesterol (TC) was estimated from serum samples by the cholesterol esterase method as reported [22], and TG was determined by the glycerol-3-phosphate oxidase method [27]. The atherogenic index (AI) was obtained from the formula given below as defined by Muruganandan, Lal, and Gupta [28]. High-density lipoprotein (HDL) cholesterol was determined on a semi-autoanalyzer using diagnostic kits (Bayer Diagnostics Ltd, Pakistan) as reported previously [14]. Low-density lipoprotein (LDL) and very low-density lipoprotein (VLDL) contents were estimated by using Friedewald's formulae.

$$\begin{array}{cc} \; & A\text{therogenic Index}=\frac{\text{Total serum cholesterol}-\text{HDL cholesterol}}{\text{HDL cholesterol}}\\ \text{LDL Cholesterol}=\text{Total serum cholesterol}-\text{HDL cholesterol}-\frac{\text{Total serum triglyceridesl}}{5}\\ \text{VLDL Cholesterol}\left(\frac{\text{mg}}{\text{dL}}\right)=\frac{\text{Total serum triglycerides}}{5} \end{array}$$

#### 2.6.2. Liver and Kidney Function Parameters in Serum

Serum alanine aminotransferase (ALT), asparate aminotransferase (AST), and bilirubin total (BT) levels in the serum were estimated to evaluate the liver function as reported earlier [25]. Serum creatinine (SC) and alkaline phosphate (AP) were measured to appraise kidney function [27]. All samples for assays were conducted spectrophotometrically by an autoanalyzer (Opera, Techicon, Bayer, United States) [29].

#### 2.6.3. Estimation of Oxidative Stress Parameters

Lipid oxidative damage in rat serum samples was evaluated by examining the lipid peroxidation product via the malondialdehyde (MDA) assay, in accordance with the methodology established by Ohkawa, Ohishi, and Yagi [30]. To investigate the enzymatic and nonenzymatic defenses against oxidative stress, glutathione (GSH) and superoxide dismutase (SOD) levels were measured in rat serum samples, employing established protocols with slight adjustments [31]. Total antioxidant capacity was measured by the procedure explained [32].

### 2.7. Statistical Analysis

For computation and the application of statistical models, the statistical package STATISTICA (StatSoft, Inc., Tulsa, OK, Oklahoma, United States) was utilized. The data obtained from various tests were subjected to analysis using a one-way analysis of variance (ANOVA), followed by the Bonferroni/Dunnett (all mean) post hoc test. Statistical significance was attributed to differences between the means if the probability value (*p*) was found to be less than or equal to 0.05.

## 3. Results

### 3.1. Extract Yield and Phenolic Profile of MPT

**Figure 1:**
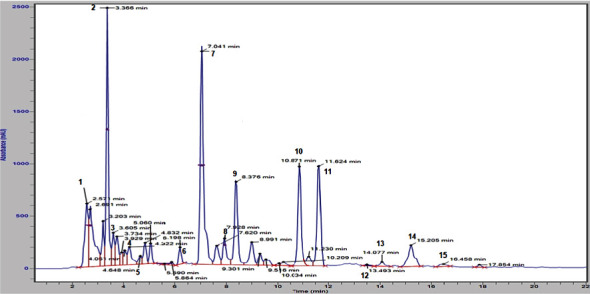
HPLC chromatogram showing the separation of polyphenols from MPT.

The MPT extract yield, based on dry plant material, was 15.22 g/100 g. Phenolic acids and flavonoids were studied by using RP-HPLC, and the data is summarized in Table 1. Thirteen phenolic acids, including gallic acid, caffeic acid, chlorogenic acid, syringic acid, p-coumaric acid, 4-hydroxy benzoic acid, vanillic acid, salicylic acid, sinapic acid, cinnamic acid, ellagic acid, ferulic acid, and benzoic acid, along with two flavonoids, namely, catechin and rutin, were quantified from the MPT (Figure 1).

Among these, salicylic acid per 100 mL MPT (71.13 mg) emerged as the primary phenolic acid detected, succeeded by gallic acid (47.78 mg), sinapic acid (45.55 mg), cinnamic acid (21.44 mg), chlorogenic acid (23.26 mg), and ellagic acid (6.90 mg). Additionally, catechin (497.0 mg/100 mL MPT) was identified as the dominant flavonoid present in the MPT, followed by rutin (447.2 mg/100 MPT).

### 3.2. Evaluation of In Vitro Antioxidant Activity

The TPC and TFC of MPT were expressed as milligrams of gallic acid and milligrams of catechin equivalent per gram of dry marigold petals, respectively (Table 2). TPC and TFC of MPT were recorded at 5.53 mg/g of dry petals and 7.73 mg/g of dry petals, respectively. Free radical scavenging activity of MPT (10 *μ*g/mL) was measured by a DPPH free radical scavenging assay. MPT solution showed 57.2% scavenging of DPPH free radical, while synthetic antioxidant BHT showed 89% scavenging (Table 2).

### 3.3. In Vivo Antiobesity Activity

#### 3.3.1. Effect of MPT on BW and Organ Weights

**Figure 2:**
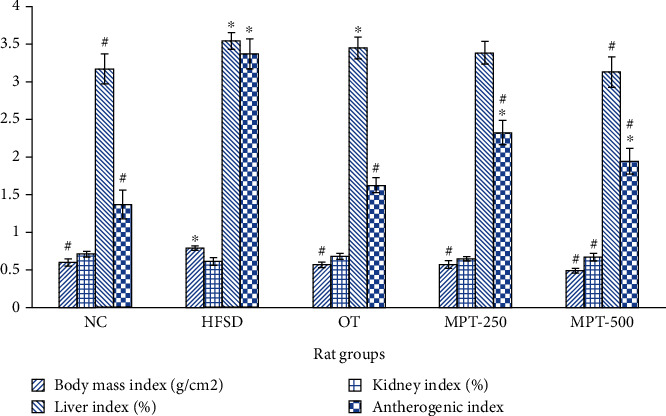
Effect of marigold petal tea and orlistat treatments on the body mass, liver, kidney, and atherogenic indices of different rat groups. Significant difference (*p* ≤ 0.05) with the NC group is presented with “∗”, and significant (*p* ≤ 0.05) difference with the HFSD group is presented with “#”.

The impact of a HFSD on the BW and BMI of rats in different groups is outlined in Table 3 and depicted in Figure 2.

Notably, the HFSD group exhibited a significant (*p* ≤ 0.05) increase in both BMI and BW when contrasted with the control group, clearly establishing the success of the induced obesity model reliant on high fat and sugar consumption. Specifically, the BMI of the HFSD-treated group measured 0.8 g/cm*^2^*, representing a statistically significant increase (*p* ≤ 0.05) in comparison to the NC group's BMI of 0.6 g/cm^2^. This group displayed a remarkable 99.3% increase in BW compared to the 40.6% increase observed in the NC group. These findings provide strong evidence of the HFSD's effectiveness in inducing obesity in the rats, as demonstrated by the considerable elevation in both BW and BMI within the HFSD group in relation to the control (NC) group. The disparities in BMI and BW between the two groups were deemed statistically significant (*p* ≤ 0.05). Notably, the application of MPT doses and the orlistat drug led to a significant (*p* ≤ 0.05) reduction in heightened BMI and BW across all treatment groups. Among these, the group administered 500 mg MPT/kg BW (MPT-500) exhibited notable effects on BMI and BW, surpassing the impact observed in the OT group. Furthermore, the study highlighted discrepancies in the weight of body organs among the different groups, particularly in the HFSD group (Table 3).

Results indicated a noteworthy increase (*p* ≤ 0.05) in kidney and liver weights within the HFSD group when compared to the NC group. Notably, higher doses of MPT contributed to a significant reduction in organ weights which were manifested by kidney and liver indices (Figure 2).

#### 3.3.2. Effect on Serum Lipid Profile

**Figure 3:**
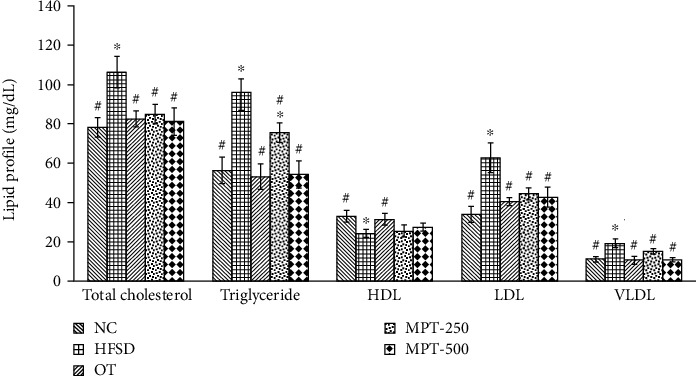
Effect of marigold petal tea and orlistat treatments on the serum lipid profiles of different groups. Significant difference (*p* ≤ 0.05) with the NC group is presented with “∗”, and significant (*p* ≤ 0.05) difference with the HFSD group is presented with “#”. Groups: NC, normal control; HFSD, high-fat-sugar diet; OT, orlistat-treated; MPT-250 and MPT-500, marigold petal tea 250 and 500 mg/kg BW; HDL, high-density lipoprotein; LDL, low-density lipoprotein; VLDL, very low-density lipoprotein.

As demonstrated in Figure 3, the HFSD group exerted a considerable effect on the serum lipid profiles. When comparing the HFSD group with the NC and treatment groups, an increase in the levels of TC, TG, LDL, and VLDL was observed while there was a decrease in the level of HDL. Treatment groups OT, MPT-250, and MPT-500 significantly (*p* ≤ 0.05) recovered the damage in terms of raised levels of serum LDL, VLDL, TC, and TG and declined levels of HDL (Figure 3). Better results were obtained with a higher dose of MPT (500 mg MPT/kg BW) with TC, TG, HDL, LDL, and VLDL values of 81.1, 54.3, 27.5, 42.7, and 10.8 mg/dL, respectively.

#### 3.3.3. Effect of MPT on Oxidative Stress Parameters

**Figure 4:**
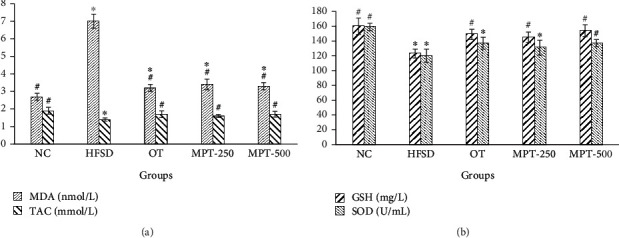
(a, b) Effect of marigold petal tea and orlistat treatments on the oxidative stress biomarkers of different groups. Significant difference (*p* ≤ 0.05) with the NC group is presented with “∗”, and significant (*p* ≤ 0.05) difference with the HFSD group is presented with “#”. Groups: NC, normal control; HFSD, high-fat-sugar diet; OT, orlistat-treated; MPT-250 and MPT-500, marigold petal tea 250 and 500 mg/kg BW; MDA, malondialdehyde; GSH, reduced glutathione; SOD, superoxide dismutase; TAC, total antioxidant capacity.

Serum levels of MDA, GSH, SOD, and TAC of NC, HFSD, OT, MPT-250, and MPT-500 groups are listed in Figure 4. The MDA level in the rat serum of the HFSD group was 7 nmol/L, whereas the GSH and SOD levels were 123 mg/L and 120 U/mL, respectively. The MDA level was higher, while GSH and SOD were lower than their levels in the NC group, which were statistically significant (*p* ≤ 0.05). The OT, MPT-250, and MPT-500 exerted positive effects by decreasing the alleviated level of MDA and increasing the suppressed levels of GSH and SOD, and these effects were statistically significant (*p* ≤ 0.05). In the GSH assay, the HFSD group has 123.2 mg/L and the NC group has 160.3 mg/L GSH levels that are significantly (*p* ≤ 0.05) varied and showed the level of oxidative stress. However, MPT effectively prevented oxidative stress in the treatment groups, and the best result was obtained in the MPT-500 group (Figures 4(a) and 4(b)). Similarly, the MPT-500 group exerted the best effect among all the treatment groups in SOD assays, and the levels of GSH and SOD in the MPT-500 group were 155 mg/L and 137 U/mL, respectively (Figures 4(a) and 4(b)). TAC of NC, OT, MPT-250, and MPT-500 in both control and treatment groups are presented in Figures 4(a) and 4(b). The TAC of the NC group was 1.9 mmol/L versus 1.36 mmol/L of the HFSD group which is significantly (*p* ≤ 0.05) lowered. Both MPT treatment groups significantly (*p* ≤ 0.05) enhanced the TAC in animal groups, and the best protective effect was recorded in the MPT-500 group (1.7 mmol/L).

#### 3.3.4. Effect of MPT on Serum Levels of Liver and Kidney Enzymes

The effect of MPT on the BT, ALT, AST, SC, and AP of rat groups is presented in Table 3. Rats fed on HFSD had significantly (*p* ≤ 0.05) increased serum levels of AST, ALT, AP, and SC, as compared to the NC group, whereas the level of BT decreased. Both treatment groups of MPT increased the BT value and decreased the concentration of ALT, AST, and AP, while no significant (*p* ≥ 0.05) effect was measured in the SC level. The maximum protective effects were noticed in the MPT-500 group which was like the PC group (Table 3).

## 4. Discussion

Phenolic compounds are abundantly found in medicinal plants and are responsible for various physiological functions. They are essential in the human diet due to their effectiveness against oxidative stress. Polyphenols are ubiquitous in plants with different concentrations. The obtained aqueous extract yield of marigolds in the present study was comparable to those previously reported in literature [33, 34]. Methanol, ethanol, and aqueous solvents are used for the isolation of polyphenols from various plant materials [35, 36]. Phenolic acids and their glycosides, aglycon, monoglycosyl, or diglycosyl flavonoids, are distributed in different solvents as a function of polarity. Owing to this, aqueous extracts contain the most polar compounds including triglycosyl flavonoids. These facts indicate the stronger scavenging property and antioxidant activity of water extracts of phenolic compounds [25]. A higher number of flavonoids was detected in flowers when compared to other plant parts, and it was mandatory to quantify the contents of these phenolic compounds to assess their contribution to antioxidant activity [36]. Methanol extract of marigold flower from Tunisia is reported to be rich in rutin and gallic acid as major flavonoids and phenolic acids [35].

TPC and TFC are usually determined to access the antioxidant potential of plant materials/extracts [35, 36]. TPC from aqueous extract of marigold petals was found to be 57.47 mg/g of dry plant material and 1.97 mg/g of extract (extract yield 23.6%) [25, 34]. However, in another study, the TFC in the aqueous extract of *C. officinalis* was found to be 40.67 mg/g of extract [25, 34]. Generally, there is an increase in DPPH free radical scavenging capacity with a proportionate increase in the dose of the extract as the phenolic contents increase [37, 38].

A HFSD exhibited an increase in BMI and BW in the present study as compared to the control group. It provides strong evidence of the HFSD's effectiveness in inducing obesity in the rats, as demonstrated by the considerable elevation in both BW and BMI within the HFSD group in relation to the control (NC) group. Although obesity depends on various factors, the dietary factor, especially the consumption of a high-calorie and high-fat diet, is regarded as one of the key threat reasons for obesity development [17]. The administration of a HFSD is known to increase BW by elevating TG levels in adipose tissue, resulting in the accumulation of fat mass [39]. In the present study, the HFSD group exhibited an increase in BW, likely due to the deposition of fat in various body fat pads resulting from the consumption of a high-calorie and high-fat diet.

The present findings were also in accordance with the study conducted by Hernández-Saavedra et al. that an aqueous extract of marigold showed antiobesity effects in rats fed a high-fat diet [39]. It is reported that a 5%–10% decrease in the BW exerted a remarkable effect on human health status [40]. However, the orlistat-treated group exhibited a lesser increase in BW, possibly because orlistat selectively reduces body fat while leaving lean body mass unchanged [41]. Catechin, a major compound identified in MPT, showed potential effects on weight loss and maintenance by promoting energy expenditure. Meanwhile, rutin, a flavonoid, is noted for its potential to reduce adiposity, increase energy expenditure, improve glucose homeostasis, and diet-induced obesity [42]. These findings mention that dietary factors, especially high-calorie and high-fat diets, can contribute to obesity development, but some MPT compounds like catechin and rutin may have potential antiobesity effects by influencing BW, fat deposition, and energy expenditure.

Monitoring of lipid profile is another important parameter when investigating the impact of herbal teas on obesity and oxidative stress. Changes in lipid profile levels observed in the HFSD group may result from factors such as enhanced intestinal fat absorption due to the activation of gastric lipase enzymes and dietary cholesterol levels. High levels of LDL and TC are correlated with a high risk of coronary heart diseases; however, HDL cholesterol is beneficial for excessive cholesterol excretion [43].

High-fat diets potentially lead to an increase in LDL cholesterol by reducing the LDL receptor active sites [44]. Therefore, a decrease in the level of LDL and TC cholesterol may be an important indicator to access the antiobesity potential in high-fat diet-fed rats. MPT was found to reduce LDL cholesterol, which may be attributed to its ability to prevent the inhibitory action of the HFD on LDL receptor sites. The results are in line with the findings of Hernández-Saavedra et al., who reported the administration of marigolds significantly decreased serum TG levels [39]. The dyslipidemia effects of MPT may be attributed to the presence of various flavonoids such as epigallocatechin. It was reported that the compound stimulated thermogenesis and decreased fat accumulation, thus inhibiting pancreatic lipase activity.

Increased levels of MDA are indicative of oxidative stress and cellular damage. GSH and SOD are also important oxidative stress biomarkers. The development of oxidative stress and cellular damage is established by increased MDA and reduced levels of SOD and GSH [45, 46] which is supported by the findings of the present study, where an increased MDA level in the HFSD group was observed. The treatment groups showed a recovery in the levels of GSH and SOD, and this may be attributed to the antioxidant potential of the polyphenols present in MPT [45]. Levels of endogenous enzymes which play a major role in the inhibition of lipid peroxidation and free radical scavenging are decreased in oxidative stress [22, 27, 45]. TAC is one of the markers of oxidative stress, and a decrease in TAC is a sign of oxidative stress [27]. Thus, an increase in the TAC level with the administration of MPT means that MPT performed well against oxidative stress.

Liver and kidney functions may be affected under obesity and oxidative stress conditions. In obesity and oxidative stress, changes in serum bilirubin (BT) levels may occur, with potential implications for heart health. Specifically, decreased BT levels are associated with abdominal obesity and correlated with certain lipid profile parameters [22, 27]. Monitoring BT levels in the context of obesity and oxidative stress may provide insights into the risk of cardiovascular diseases and the functioning of the liver and kidneys [47]. Jenko-Pražnikar et al. and Chang et al. reported that the serum BT level decreased in the abdominal obesity condition and was associated with the levels of LDL, TC, and TG [48, 49].

## 5. Conclusions

Current study explored that MPT is a potential source of phenolic acids and flavonoids. Among the phenolic acids, salicylic acid was found to be the most abundant, while catechin emerged as the major flavonoid present. In vitro antioxidant testing demonstrated that MPT possesses antioxidant and DPPH free radical scavenging properties. In vivo analysis revealed that the administration of MPT at doses of 250 and 500 mg/kg BW exhibited beneficial effects in countering oxidative stress, high cholesterol levels, and obesity in a dose-dependent manner.

## Figures and Tables

**Table 1:** Contents of phenolic acids and flavonoids identified from MPT by RP-HPLC.

**Compounds**	**Retention time (min)**	**Concentration (mg/100 mL of MPT)**
1. Gallic acid	2.571	47.78 ± 2.30
2. Catechin	3.366	497.0 ± 19.3
3. Chlorogenic acid	3.605	23.26 ± 1.20
4. Caffeic acid	4.222	5.95 ± 0.31
5. Syringic acid	4.648	2.14 ± 0.10
6. *p*-Coumaric acid	6.198	4.31 ± 0.20
7. 4-Hydroxybenzoic acid	7.041	9.85 ± 0.50
8. Vanillic acid	7.928	6.57 ± 0.31
9. Salicylic acid	8.376	71.13 ± 3.01
10. Rutin	10.871	447.2 ± 20.1
11. Sinapic acid	11.624	45.55 ± 2.11
12. Ferulic acid	13.493	0.92 ± 0.05
13. Ellagic acid	14.077	16.90 ± 0.92
14. Cinnamic acid	15.205	21.44 ± 1.01
15. Benzoic acid	16.458	1.09 ± 0.06

*Note:* The values are reported as mean ± standard deviation of three independent experiments.

**Table 2:** Total phenolic contents, total flavonoid contents, and DPPH radical scavenging activity of MPT.

**Assays**	**MPT**	**BHT**
TPC (milligrams per gram of dry plant material, measured as gallic acid equivalent)	5.53 ± 0.17	—
TFC (milligrams per gram of dry plant material, measured as catechin equivalent)	7.73 ± 0.68	—
DPPH radical scavenging activity (percentage) exhibited by 10 *μ*g/mL solution	57.2 ± 1.9^a^	89.3 ± 4.3^b^

*Note:* The values are reported as mean ± standard deviation of three independent experiments. Different alphabets (^a, b^) in superscript in the same row represent significant (*p* ≤ 0.05) differences among MPT and BHT.

**Table 3:** Effect of marigold petal tea and orlistat on the body, kidney and liver weights, liver, and kidney parameters of different groups of obesity rat model.

**Groups**	**Body weight**			**Liver parameters**				**Kidney parameters**		
	**Initial (g)**	**Final (g)**	**%increase**	**Weight (g)**	**BT (mg/dL)**	**AST (*μ*/L)**	**ALT (*μ*/L)**	**Weight (g)**	**AP (*μ*/L)**	**SC (mg/dL)**
NC	155 ± 13	218 ± 10^$^	40.6	6.92 ± 1.11^#^	0.4 ± 0.05^#^	63 ± 5^#^	62 ± 4^#^	1.56 ± 0.24	143 ± 9^#^	0.4 ± 0.1
HFSD	143 ± 17	285 ± 14^$^	99.3	10.10 ± 1.00^∗^	0.3 ± 0.02^∗^	98 ± 5^∗^	81 ± 5^∗^	1.74 ± 0.17	164 ± 9^∗^	0.6 ± 0.2
OT	145 ± 11	228 ± 21^$^	57.2	8.13 ± 1.13	0.4 ± 0.03^#^	63 ± 6^#^	62 ± 4^#^	1.57 ± 0.23	152 ± 8	0.4 ± 0.1
MPT-250	148 ± 11	251 ± 16^$^	69.6	7.52 ± 1.09^#^	0.4 ± 0.03^#^	77 ± 4^∗^^#^	73 ± 3^∗^	1.62 ± 0.22	148 ± 9	0.4 ± 0.1
MPT-500	143 ± 12	217 ± 15^$^	51.74	5.93 ± 1.05^#^	0.5 ± 0.04^#^^∗^	63 ± 5^#^	63 ± 3^#^	1.58 ± 0.22	143 ± 6	0.4 ± 0.1

*Note:* The values are reported as mean ± standard deviation of six rats of the same group. Different symbols in superscripts in the same column showed significant (*p* ≤ 0.05) differences among different groups.

Abbreviations: ALT, alanine aminotransferase; AP, alkaline phosphatase; AST, aspartate aminotransferase; BT, bilirubin total; HFSD, high-fat-sugar diet; MPT-250 and MPT-500, marigold petal tea 250 and 500 mg/kg BW; NC, normal control; OT, orlistat-treated; SC, serum creatinine.

^$^Exhibited significant (*p* ≤ 0.05) increase in the final body weight compared to the initial body weight of all groups. Significant (*p* ≤ 0.05) difference of the treatment group compared to the NC group.

^#^Significance (*p* ≤ 0.05) of the treatment groups compared to the HFSD group.

## Data Availability

All the data is provided in this manuscript as tables and figures.
